# The global burden of premature mortality due to the Middle East respiratory syndrome (MERS) using standard expected years of life lost, 2012 to 2019

**DOI:** 10.1186/s12889-019-7899-2

**Published:** 2019-11-14

**Authors:** Maryam Salamatbakhsh, Kazhal Mobaraki, Sara Sadeghimohammadi, Jamal Ahmadzadeh

**Affiliations:** 1 MSc of Critical Care Nursing, Urmia University of Medical Sciences, Urmia, Iran; 2 Epidemiologist, Social Determinants of Health Research Center, Urmia University of Medical Sciences, Urmia, Iran; 3 MSc of Critical Care Nursing, School of Nursing and Midwifery, Zanjan University of Medical Sciences, Zanjan, Iran

**Keywords:** Standard expected years of life, Premature mortality, Burden of disease, Worldwide

## Abstract

**Background:**

It has been 8 years since the first case of Middle East respiratory syndrome coronavirus (MERS-CoV) was reported in Saudi Arabia and the disease is still being reported in 27 countries; however, there is no international study to estimate the overall burden related of this emerging infectious disease. The present study was conducted to assess the burden of premature mortality due to Middle East respiratory syndrome (MERS) worldwide.

**Methods:**

In this retrospective analysis, we have utilized publicly available data from the WHO website related to 1789 MERS patients reported between September 23, 2012 and May 17, 2019. To calculate the standard expected years of life lost (SEYLL), life expectancy at birth was set according to the 2000 global burden of disease study on levels 25 and 26 of West model life tables from Coale-Demeny at 82.5 and 80 years for females and males, respectively.

**Results:**

Overall, the total SEYLL in males and females was 10,702 and 3817.5 years, respectively. The MERS patients within the age range of 30–59 year-olds had the highest SEYLL (8305.5 years) in comparison to the patients within the age groups 0–29 (SEYLL = 3744.5 years) and ≥ 60 years (SEYLL = 2466.5 years). The total SEYLL in all age groups in 2012, 2013, 2014, 2015, 2016, 2017, 2018, and 2019 were 71.5, 2006.5, 3162, 4425.5, 1809.5, 878, 1257.5 and 909 years, respectively. The most SEYLL related to MERS-CoV infection was in the early four years of the onset of the pandemic (2012 to 2015) and in the last four years of the MERS-CoV pandemic (216 to 2019), a significant reduction was observed in the SEYLL related to MERS-CoV infection in the MERS patients.

**Conclusion:**

We believe that the findings of this study will shed light about the burden of premature mortality due to MERS infection in the world and the results may provide necessary information for policy-makers to prevent, control, and make a quick response to the outbreak of MERS-CoV disease.

## Background

Various indexes of premature death are proposed according to years of life lost. Standard expected years of life lost (SEYLL) is a valid measure that is widely used for prioritizing and identifying the causes of premature death [1, 2]. The SEYLL as one component of the disability-adjusted life years (DALYs) is used to emphasize premature mortality by estimating the average years a person would have lived if he or she had not died prematurely [3, 4].

In order to determine and estimate the relative importance of the different causes of death and the extent of public health problems, other epidemiological mortality indexes such as percentage of case fatality rate (CFR%), disease or cause-specific mortality rate, and proportionate mortality are used frequently [5]. Despite their usefulness, they often fail to calculate the overall burden of premature mortality related to a common and rare disease in certain populations [6].

The SEYLL approach has been used by most of the studies about the burden of the disease up to now [1, 7, 8], but to the best of our knowledge, previous studies have neglected or had not estimated the burden of premature mortality for Middle East respiratory syndrome coronavirus (MERS-CoV) infection. In the last decade, we have seen the emergence of coronaviruses, which are responsible for acute respiratory infections with a high mortality rate [9].

MERS-CoV is an emerging novel beta coronavirus belonging to lineage C and the first case of this infection was reported in 2012 in Jeddah, Saudi Arabia [10, 11]. Since then, 27 countries were affected by MERS-CoV and have reported MERS cases to the world health organization (WHO) [9, 12].

Policy-makers face the challenge over-controlling and preventing the disease, since all of the decisions must be made according to summary indexes that quantify the burden of disease at the population level including SEYLL. Furthermore, previous studies on burden of premature mortality [1, 8, 13, 14] based on SEYLL were mostly from a single population. In the present study, in addition to recalling the importance of using SEYLL to estimate the burden of premature mortality for emerging diseases, we for first time present the estimates of the global burden of premature mortality in laboratory-confirmed MERS-CoV cases.

## Methods

We retrieved the data related to laboratory-confirmed MERS-CoV cases from September 23, 2012 until May 17, 2019 for this retrospective analysis from the disease outbreak news in the WHO website:(http://www.who.int/csr/don/archive/disease/coronavirus_infections/en/). The WHO received all the case reports (describing details case-by-case) of MERS-CoV in a narrative format on behalf of the national-international health regulation focal point of 27 countries of the world. Data will be updated periodically on the mortality and the incidence of the MERS cases. We have retrieved the main epidemiologic information of each MERS patient.

To calculate SEYLL(without discounting or age-weighting), we categorized the dataset related to MERS cases into 3-year age groups based on each person’s age at the time of death. Then, the age at the time of death for each individual in the mentioned age groups were subtracted from the relevant reference age using the following equation:

$$ \mathrm{SEYLL}=\sum \left({\mathrm{Ni}}^{\mathrm{m}}\times {\mathrm{Li}}^{\mathrm{m}}+{\mathrm{Ni}}^{\mathrm{f}}\times {\mathrm{Li}}^{\mathrm{f}}\right) $$

where Ni^m^ (Ni^f^) represents the number of male (female) deaths in the age group i, multiplied by the respective standard life expectancy, Li^m^(Li^f^) based on the 2000 Coale-Demeny life table with life expectancy of 80 years for men and 82.5 years for women [15].. The SEYLL for each individual were then added together to estimate the total SEYLL for all MERS patients who died in a particular year. Finally, we calculated the SEYLL for all age-groups and both genders by cause of mortality and reported these as percentages. All the statistical analysis were conducted using the SPSS, version 21 (IBM Inc., Armonk, NY, USA). Quantitative and qualitative measures were expressed by mean ± S.D. and absolute frequencies and percentages, respectively. Chi-square test was used to determine the frequency distributions between probable risk factors and the final outcome (dead/alive) of the laboratory-confirmed MERS-CoV cases in Table 1. Any *p*-value given was two-sided and was considered statistically significant at 0.05.

**Table 1:** Sample characteristics and distribution of deaths among 1789 MERS patients, September 23, 2012 until May 17, 2019

Characteristics	Levels	Total (%) (*n* = 1789)	Death (*n* = 588)	Alive (*n* = 1201)	*P*-value
	≤ 9	8(0.4)	5	3	
	10–19	27(1.5)	8	19	
	20–29	314(17.6)	52	262	
	30–39	215(12.0)	63	152	
Age, yr	40–49	264(14.8)	76	188	< 0.001
	50–59	321(17.9)	103	218	
	60–69	333(18.6)	114	219	
	70–79	214(12.0)	118	96	
	≥ 80	93(5.2)	49	44	
Sex	Female	494(27.6)	145	349	0.028
	Male	1295(72.4)	443	852	
	Saudi Arabia	1517(84.7)	480	1037	
	United Arab Emirates	87(4.9)	37	50	
	Republic of Korea	57(3.2)	10	47	
Reporting country	Oman	25(1.4)	9	16	< 0.001
	Jordan	26(1.5)	22	4	
	Qatar	26(1.5)	8	18	
	Others	51(2.9)	22	29	
Exposure with a morbid case in the previous 14 days	Yes	642(35.5)	218	424	0.248
	No	1147(64.1)	370	777	
Exposure to a camel in 14 days ago	Yes	842(47.1)	206	636	< 0.001
	No	947(52.9)	382	565	
Travel history to an endemic country	Yes	433(24.2)	141	292	0.463
	No	1356(75.8)	447	909	
Admission in hospital	Yes	1488(83.2)	515	973	< 0.001
	No	301(16.8)	73	228	
Need for admission in negative. pressure isolate room or ICU	Yes	1145(64.0)	370	775	0.270
	No	644(36.0)	218	426	
Health care personnel	Yes	277(15.5)	46	231	< 0.001
	No	1512(84.5)	542	970	
Comorbidities	Yes	1088(60.8)	327	761	0.002
	No	701(39.2)	261	440	

## Results

For the present study, data related to a total of 1789 MERS cases (1517 from Saudi Arabia, 87 from United Arab Emirates, 57 from Republic of Korea, 25 from Oman, 26 from Jordan, 26 from Qatar, and 51 from other 21 countries) including 558 deaths (the overall %CFR of the pandemics with MERS-CoV was 31.1% [558/1789]) with complete data were used in the analysis. The %CFR for 2012, 2013, 2014, 2015, 2016, 2017, 2018, and 2019 were 55.5% [5/9], 61.0% [72/118], 50.2% [96/191], 32.7% [193/590], 39.4% [77/195], 18.6% [43/230], 30.3% [62/305], and 26.4% [40/151], respectively.

The descriptive results on demographic characteristics of the population can be found in Table 1. The MERS patients had a mean age of 50.6 (±S.D. 18.4 years), with an age range of between 2 and 109 years. Most of the global MERS cases were within the age range of 20–79 years old (92.8% [1661/1789]). Males (72.4% [1295/1789]) seem to be more affected than females (27.6% [494/1789]). About 83.2% [1488/1789] MERS cases were admitted to hospital and among them, 64% [1145/1789] needed admission in negative pressure isolate room or ICU. Also, 60.8% [1088/1789] MERS cases had one or more comorbidity (*p* > 0.05 for all).

Table 2 illustrates the distribution of deaths and SEYLL by age groups and year during the course of this study. In total, data shows that the absolute frequency of deaths associated with MERS-CoV infection in males was higher than females in all three age groups (more than twice). Also, MERS cases with ≥60 years had a higher absolute frequency of deaths in comparison to those in age groups 0–29 and 30–59 years olds. Based on the analysis, in all three age groups, the SEYLL index in males was 10,702 years and in females, this index was 3817.5 years. The MERS patients with 30–59 year-olds had the highest SEYLL (8305.5 years) in comparison to those in age groups 0–29 (SEYLL = 3744.5 years) and ≥ 60 years (SEYLL = 2466.5 years). The total SEYLL in both genders and in all age groups in 2012, 2013, 2014, 2015, 2016, 2017, 2018, and 2019 were 71.5, 2006.5, 3162, 4425.5, 1809.5, 878, 1257.5, and 909 years, respectively.

**Table 2:** Distribution of deaths and SEYLL among 588 MERS cases by age groups and year (September 23, 2012 until May 17, 2019)

Year	Age Groups (year)																	
	0–29 yr (*n* = 65)						30–59 yr (*n* = 242)						≥60 yr (*n* = 281)					
	Female			Male			Female			Male			Female			Male		
	Deaths	SEYLL	SEYLL%	Deaths	SEYLL	SEYLL%	Deaths	SEYLL	SEYLL%	Deaths	SEYLL	SEYLL%	Deaths	SEYLL	SEYLL%	Deaths	SEYLL	SEYLL%
2012 *n* = 5	0	0	0.0	0	0	0.0	0	0	0.0	0	0	0.0	3	45.5	5.6	2	26	1.6
2013 *n* = 72	2	111	9.9	8	504	19.2	10	378	20.1	19	689	10.7	11	130.5	16.1	22	194	11.7
2014 *n* = 96	5	285.5	25.4	21	1155	44.1	10	386	20.5	30	1019	15.9	9	74.5	9.2	21	242	14.6
2015 *n* = 193	7	431.5	38.4	9	494	18.8	21	771.5	41.0	53	1927	30.0	27	289.5	35.7	76	512	30.9
2016 *n* = 77	3	166.5	14.8	1	56	2.1	5	152.5	8.1	38	1171	18.2	3	27.5	3.4	27	236	14.3
2017 *n* = 43	0	0	0	3	169	6.4	2	73	3.9	11	336	5.2	18	207	25.5	9	93	5.6
2018 *n* = 62	1	67.5	6.0	3	191	7.3	1	37.5	2.0	24	723	11.3	1	5.5	0.7	32	233	14.1
2019 *n* = 40	1	60.5	5.4	1	53	2.0	2	85	4.5	16	560	8.7	3	31.5	3.9	17	119	7.2
Total n = 588	19	1122.5	100	46	2622	100	51	1883.5	100	191	6425	100	75	811.5	100	206	1655	100

**Fig. 1:**
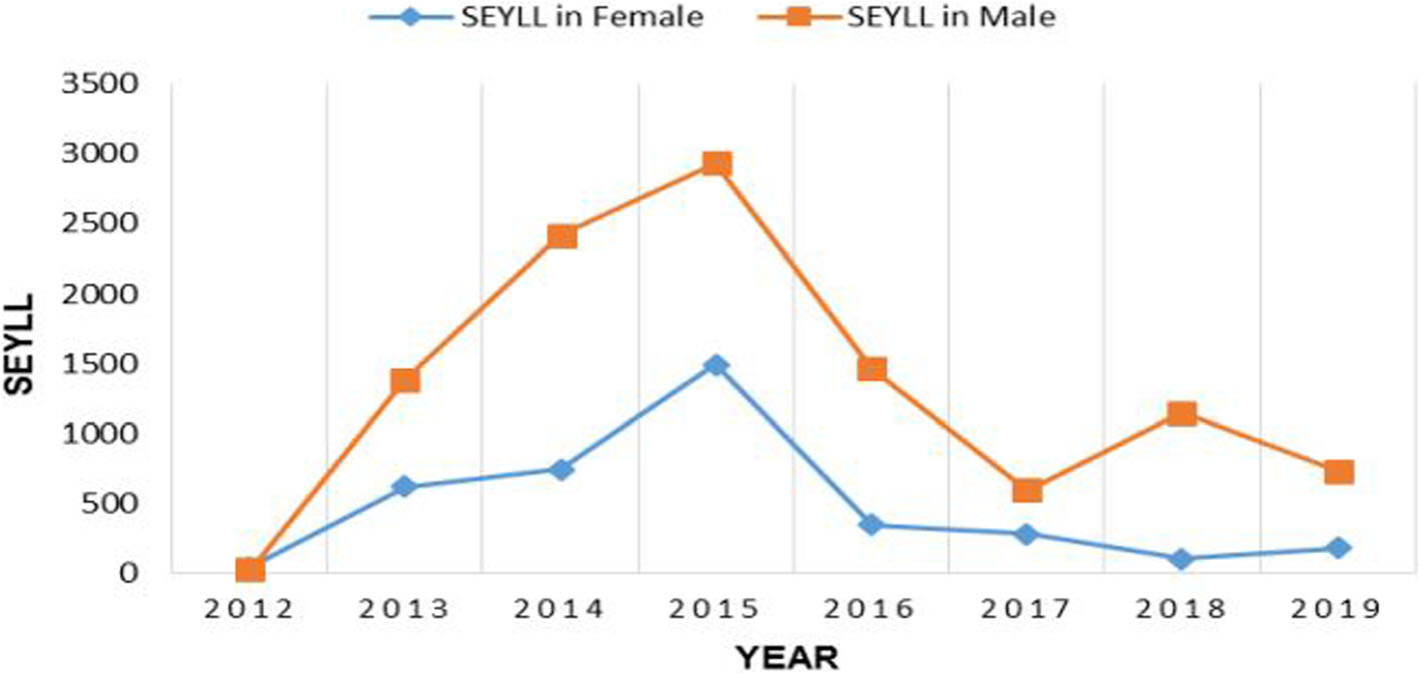
The trend of SEYLL by year and sex in all involved countries in the world, September 23, 2012 until May 17, 2019

Figure 1 shows the trend of SEYLL by year and sex in all involved countries in the world from September 23, 2012 until May 17, 2019.

## Discussion

As noted in some of the previous articles [6, 13, 16, 17], the estimation of the SEYLL not only plays an important role in the evaluation of the extent of public health problems, but also in estimating the social and economic loss. Indeed, the commonly used SEYLL enables the severity of the specific infection and also the relative importance of the different causes of death to be determined. The results of this study indicate that after 8 years since the start of MERS-CoV pandemic, the overall %CFR associated with it is still high (31.1%). Perhaps one of the reasons for this is the lack of a vaccine or specific treatment for fighting against this infection till now and all treatments are supportive and often they are based on the patient’s clinical condition.

Our finding shows that 60.8% of MERS patients had one or more comorbidity. Most of the previously published studies of MERS-CoV declared that comorbidities could have a substantial impact on MERS prognosis and severity of disease [12, 18–20]. Until more is understood about MERS-CoV infection, we believe that patients with diabetes mellitus, hypertension, ischemic heart disease, and end-stage renal diseases should be considered at a higher risk for MERS-CoV infection and in the triage, these patients seem to be prioritized for treatment.

The available data indicates that in all age groups, male MERS cases had a higher SEYLL (10,702 years) in comparison to the female MERS cases (3817.5 years). Previous research has demonstrated a significant difference in mortality related to MERS between men and women [9, 21].. In line with our finding, Maniecka-Bryla et al. found that men diagnosed with the respiratory system disease in comparison to women had a higher SEYLL [22]. We would like the SEYLL to decrease over time; however, with a bird’s-eye view to Fig. 1, this pattern is changing since mid-2018 and the burden of MERS-CoV infection in MERS patients has begun to increase in women. For the men at the same time, the downward trend of SEYLL associated with MERS-CoV infection had continued. The findings in the current study can be considered as a warning to public health authorities.

We also found that MERS patients within the age group 30–59 year-olds had the highest SEYLL (8305.5 years) in comparison to those in age groups 0–29 (SEYLL = 3744.5 years) and ≥ 60 years (SEYLL = 2466.5 years). This important issue that individuals in active age of their life had the highest SEYLL should be considered by health policy-makers in implementation of appropriate prevention and control measures to reduce the burden of premature mortality due to MERS CoV in the affected populations. Furthermore, the values of SEYLL indicate that diseases of the respiratory system play an important socio-economic role in the health status of the population worldwide.

According to the data (Fig. 1), the trend of SEYLL by year and sex in all involved countries in the world from September 23, 2012 to May 17, 2019 has decreased. It is apparent that the SEYLL values were higher in men than women for all years of the study. Overall, the highest SEYLL related to MERS-CoV infection was in the early four years of the onset of the infection (2012 to 2015) and then in mid-2015 the SEYLL in MERS cases declined rapidly and in the last four years of the MERS-CoV pandemic (216 to 2019), there has been a significant reduction in the burden of mortality related to MERS-CoV infection in morbid cases. In the literature review, we did not find any studies comparing these indices, however, this pattern was also reported during the epidemic of the severe acute respiratory syndrome (SARS) [23, 24]. The remarkable reduction in the burden of premature mortality due to MERS-CoV indicates the technical guidance on surveillance of MERS patients, also suggests standardized approach and activities for health care providers to control and prevent MERS-CoV in involved countries. Although, these measures by WHO and other related national and international organizations should continue.

The current study suffered from some limitations. Of the total cases worldwide (Since September 2012, WHO has been notified of 2449 laboratory-confirmed cases of infection with MERS-CoV.), only the details of 1789 cases were investigated in the current study. It should be noted that of 186 MERS-CoV cases in the Republic of Korea, only details related to 57 cases were published in the disease outbreak news on the WHO website. The lack of complete details for all MERS cases potentially increases the occurrence of the selection and measurement bias in the results. Another limitation of this study is choosing a life expectancy for the SEYLL measure. This index does not take into account the biological and genetic differences between men and women.

In the future, we may assess the burden of premature mortality due to the MERS-CoV infections globally to better understand the risks of this new infection for public health and also to provide a helpful recommendation for controlling and preventing it. Recommendations might change and be updated as the additional data becomes available. Indeed, despite the above limitations, such studies might be useful to implement the educational programs, to access the health care and early awareness of changes in the pattern of this relatively new infection, to reduce the higher mortality rates not only for MERS-CoV but also for the other emerging pathogens in the worldwide.

## Conclusion

The analysis of SEYLL related to MERS infection in the current study presents this important issue that the loss resulting from the premature mortality has social and economic implications. These findings are also useful for policy-makers because the infection prevention and control measures are critical to prevent the possible spread of MERS-CoV infection in the affected communities.

## Data Availability

The dataset used and/or analyzed during the current study are available from the corresponding author on reasonable request.

## Abbreviations

%CFR: Percentage of case fatality rate
MERS-CoV: the Middle East respiratory syndrome coronavirus
OR: Odds ratio
S.D.: Standard deviation
SARS: Severe acute respiratory syndrome
UAE: United Arab Emirates
WHO: World Health Organization
